# POC Sensor Systems and Artificial Intelligence—Where We Are Now and Where We Are Going?

**DOI:** 10.3390/bios15090589

**Published:** 2025-09-08

**Authors:** Prashanthi Kovur, Krishna M. Kovur, Dorsa Yahya Rayat, David S. Wishart

**Affiliations:** 1 Department of Biological Sciences, University of Alberta, Edmonton, AB T6G 2E9, Canada; kovur@ualberta.ca (P.K.); kkovur@ualberta.ca (K.M.K.); dorsa.yahya@ualberta.ca (D.Y.R.); 2 The Metabolomics Innovation Centre (TMIC), Edmonton, AB T6G 2E9, Canada; 3 Department of Computing Sciences, University of Alberta, Edmonton, AB T6G 2E8, Canada; 4 Department of Laboratory Medicine and Pathology, University of Alberta, Edmonton, AB T6G 2B7, Canada; 5 Faculty of Pharmacy and Pharmaceutical Sciences, University of Alberta, Edmonton, AB T6G 2H7, Canada

**Keywords:** point-of-care devices, machine learning, artificial intelligence, predictive analytics, real-time decision support, personalized healthcare, wearable medical technology, automation in diagnostics

## Abstract

Integration of machine learning (ML) and artificial intelligence (AI) into point-of-care (POC) sensor systems represents a transformative advancement in healthcare. This integration enables sophisticated data analysis and real-time decision-making in emergency and intensive care settings. AI and ML algorithms can process complex biomedical data, improve diagnostic accuracy, and enable early disease detection for better patient outcomes. Predictive analytics in POC devices supports proactive healthcare by analyzing data to forecast health issues and facilitating early intervention and personalized treatment. This review covers the key areas of ML and AI integration in POC devices, including data analysis, pattern recognition, real-time decision support, predictive analytics, personalization, automation, and workflow optimization. Examples of current POC devices that use ML and AI include AI-powered blood glucose monitors, portable imaging devices, wearable cardiac monitors, AI-enhanced infectious disease detection, and smart wound care sensors are also discussed. The review further explores new directions for POC sensors and ML integration, including mental health monitoring, nutritional monitoring, metabolic health tracking, and decentralized clinical trials (DCTs). We also examined the impact of integrating ML and AI into POC devices on healthcare accessibility, efficiency, and patient outcomes.

## 1. Introduction

Point-of-care (POC) devices are portable platforms that provide medical diagnostics directly at the bedside. They have become indispensable for rapid clinical decision-making, particularly when access to centralized laboratories is limited [1,2,3,4,5,6,7,8,9,10,11,12,13]. The lineage of POCs can be traced back to 1965, when Dextrostix blood-glucose testing strips were first introduced. This early qualitative POC colorimetric test was initially used only by physicians and required manual comparisons to printed color charts. The first automated POC blood glucose meter, the Ames Reflectance Meter, was introduced in 1970 to quantify Dextrostix readouts in hospitals and clinics. By 1980, the Dextrometer was launched, which used Dextrostix along with a digital display to support at-home blood glucose monitoring [14]. Since then, POC devices have evolved into highly integrated microfluidic and/or wearable platforms that can perform far more than just blood glucose monitoring. Many can perform multiplexed measurements of dozens of biomarkers while demanding only microliter sample volumes [15,16,17].

Accelerated by breakthroughs in miniaturization, photolithography, and additive manufacturing, these modern POC devices embody a broader trend toward personalized data-driven medicine, providing far more equitable diagnostic access to both resource-abundant and resource-limited settings. POC devices not only democratize medical diagnostics but also reduce costly infrastructural burdens. At the heart of this transformation are several convergent engineering innovations, including lab-on-chip (LoC) and lab-on-printed-circuit-board (LoPCB) concepts, as well as innovative signal transduction modalities, novel lateral flow concepts, and new nanomaterials. LoC and LoPCB systems integrate sample preparation, reaction chemistry, and detection onto postage-stamp-sized substrates. This shrinks assay times from hours to minutes and reagent consumption by orders of magnitude [18,19]. Diverse transduction modalities have matured in parallel: electrochemical sensors yield attomolar detection limits with minimal power draw, optical schemes harness fluorescence or plasmonics for highly specific multiplexed readouts, and paper-based lateral flow assays exploit capillary flow for low-cost disposable diagnostics ideally suited to field deployment [20]. Nanomaterials, such as carbon nanotubes, quantum dots, and graphene, further amplify the surface reactivity and signal-to-noise ratio, pushing sensitivity into the sub-picomolar domain while enabling POC devices to have flexible form factors [21].

Collectively, these advances underpin a new generation of POC platforms that rival, and in certain cases surpass, the analytical performance of standard benchtop assays. The clinical adoption of POC systems has been swift, wherever rapid actionable data confer a survival benefit or streamline chronic disease surveillance. For example, POC devices that measure and interpret electrophysiological signals, such as electrocardiograms (ECGs), electroencephalograms (EEGs), electromyograms (EMGs), and photoplethysmograms (PPGs), have become widely used as they facilitate quick, accessible, and often real-time health evaluations outside of traditional labs or hospital environments, either at or near the location of patient care [22]. In oncology, serum protein or exosome panels measured on portable electrochemical chips are edging toward the sensitivity required for routine early cancer screening [23]. Diabetology continues to benefit from minimally invasive POC glucose sensors that enable tight glycemic control [24], while cardiovascular care leverages POC troponin-responsive cartridges to triage chest pain patients in emergency departments [25]. Beyond disease detection, continuous monitoring via POC epidermal sweat or interstitial fluid sensors is now being used in training regimens in sports medicine to guide personalized rehabilitation trajectories [26].

Coupled with these POC sensor breakthroughs, recent advances in machine learning (ML) and artificial intelligence (AI) have transformed POCs by elevating diagnostic accuracy, accelerating time-to-result, and ultimately improving patient outcomes [27,28,29]. By detecting subtle patterns often overlooked by human observers, AI facilitates earlier and more reliable diagnoses [30], thereby expanding access to quality care in remote or resource-constrained settings [31,32,33] and supporting underserved populations through decentralized testing [17,34]. Integrated AI also enables complex data analytics, predictive modeling, and personalized therapeutic strategies that extend beyond the traditional POC capabilities [29].

While the terms AI and ML are often used interchangeably, it is important to remember that AI refers to the broader field of simulating human-like intelligence in machines, whereas ML is a subset of AI focused on enabling systems to learn patterns from data. AI is a term that has more public visibility and is more widely used in the popular press, but for the purposes of this review, we will primarily focus on ML. This is because nearly all of the AI innovations in POC applications have been via ML and ML-related algorithms.

Over the past decade, both traditional and more advanced ML approaches have been widely applied to large, heterogeneous, medically derived datasets to generate accurate, actionable insights essential for early disease detection, precision diagnosis, and personalized medicine [35]. For example, ML methods have demonstrated the ability to classify dermatological lesions with an accuracy comparable to that of board-certified dermatologists [36]. Similar ML approaches have also been used in pathology to markedly accelerate cancer cell identification and reduce observer variability [37]. In critical-care environments, ML models that analyze time-series data from electronic health records (EHRs), vital signs, and laboratory results have been successfully applied to predict outcomes, detect patient deterioration, and recommend interventions [38,39].

**Figure 1:**
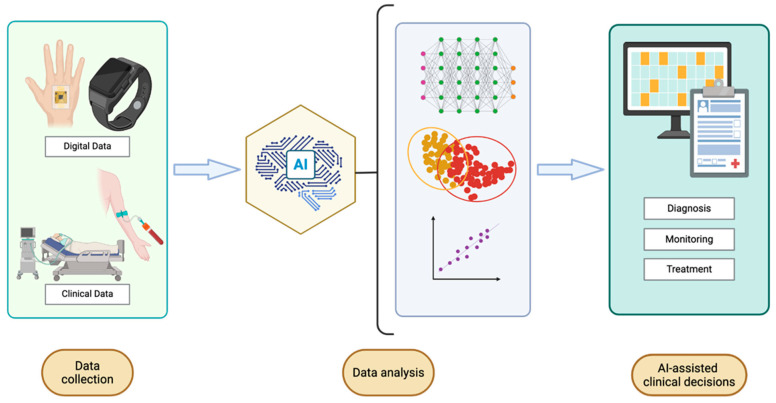
Schematic representation of ML integration into POC sensor systems. Data collection includes both digital signals from wearable or implantable sensors and clinical data from traditional diagnostics. These multimodal data are processed and analyzed using ML algorithms such as classification, clustering, and regression models. The resulting outputs support clinical decision-making by enabling early diagnosis, continuous monitoring, and personalized treatment recommendations.

ML can be divided into three classes: (1) supervised learning, which uses labelled data to make predictions; (2) unsupervised learning, which uncovers patterns in unlabelled data; and (3) reinforcement learning (RL), in which agents learn optimal actions through trial and error in dynamic environments based on reward feedback. Supervised learning is particularly useful for medical imaging applications, as it relies on labeled datasets, such as annotated radiographs, to deliver rapid, automated interpretation for early diagnosis and therapy planning [40]. Predictive analytics (another form of supervised learning) leverages labeled historical data and advanced ML-based regression methods to proactively identify high-risk individuals to enable timely preventive care, lower readmission rates, and curtail overall healthcare expenditures [29,41,42]. Conversely, unsupervised approaches are best used to interrogate large collections of unlabeled patient records, uncovering latent phenotypes or disease subtypes that inform precision treatment without prior label knowledge [43]. RL models are best used to refine decision rules through iterative feedback, offering clinicians evidence-based guidance for complex treatment plans [44]. These ML innovations are transforming POC platforms from simple diagnostic tools to sophisticated integrated clinical solutions that combine multimodal sensor data with ML analytics. By fusing data from wearables, implantables, and clinical sources, ML algorithms can detect patterns and generate predictive insights that enable early diagnosis, continuous monitoring, and personalized treatment. Figure 1 illustrates how ML enhances POC systems through multimodal data acquisition and algorithmic processing to support proactive healthcare delivery.

**Figure 2:**
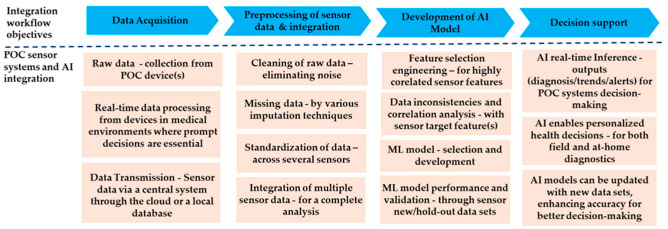
Schematic diagram depicting the integration workflow from data acquisition to decision support for POC sensor systems and AI.

Figure 2 presents a schematic workflow showing how POC device data are being integrated with AI. It illustrates the pathway beginning with data acquisition from sensors, moving through ML-based processing and analysis, and concluding in clinical decision support.

This review surveys the current landscape of ML-enabled POC devices, emphasizing the advances in data analytics, pattern recognition, real-time decision support, predictive modeling, workflow automation, and personalization. Representative examples include smart glucose monitors, ML-enabled portable imaging platforms, ML-assisted wearable cardiac monitors, ML-enhanced rapid infectious-disease tests, and smart wound care sensors, which are discussed in detail. Emerging frontiers such as ML-enabled mental health monitoring, nutritional and metabolic assessments, and decentralized clinical trials (DCTs) are also highlighted, underscoring the rapidly expanding scope of ML integration within POC biosensing.

## 2. Key Areas of ML and AI Integration in POC Devices

**Figure 3:**
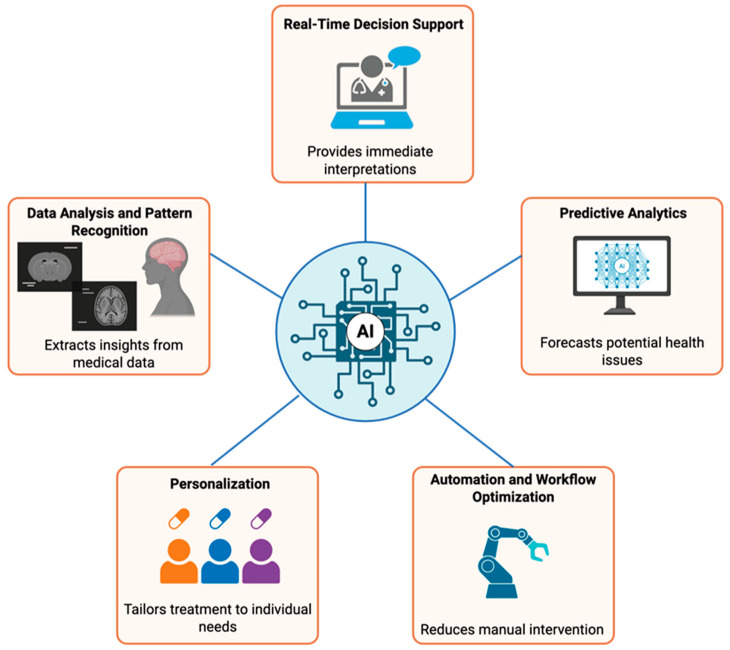
Integration of diverse biosensing platforms with AI and ML for health monitoring. The convergence of various sensor technologies, including electrochemical sensors, wearable sensors, optical sensors, and microfluidic devices with ML for data processing and interpretation. The integration enables real-time, intelligent analysis of physiological and biochemical signals for personalized health diagnostics and monitoring.

Machine learning (ML) technologies use data from modern biosensors to provide refined analysis, clinical decision support, predictive insights, and automated workflow (Figure 3). In this section, we examine how ML methodologies have been integrated into point-of-care (POC) devices, reviewing algorithms and applications, while discussing the POC sensors that are most adaptable to these technologies.

### 2.1. Data Analysis and Pattern Recognition

The critical capacity of ML in POC devices is to discern patterns in complex diagnostic data streams imperceptible through traditional analysis. In biomedical contexts, these data include physiological signals such as electrocardiograms (ECGs), glucose monitoring time-series data, and medical imaging (X-ray, CT, ultrasound, etc.). Classical ML algorithms include decision trees (DTs), random forests (RFs), support vector machines (SVMs), and simplified artificial neural networks (ANNs). These methods have fixed architectures, limited reasoning depth, and are restricted to simpler pattern recognition tasks. Advanced deep learning (DL), a subfield of ML, uses complex multi-layered neural networks such as convolutional neural networks (CNNs), recurrent neural networks (RNNs), and transformer-based models for higher-dimensional, greater reasoning depth, and more advanced pattern analysis or image recognition tasks [35]. One of the key ML techniques is CNNs, which capture spatial and temporal features in sensor input. CNNs can be used for image extraction and identification of pathological biomarkers in dermatology and radiology, as well as in the interpretation of spectral glucose signatures. Another class of ML algorithms is RNNs, which are designed to handle sequential data by modeling temporal dependencies. CNNs and RNNs enable robust image and time-series data interpretation across a wide range of POC modalities, including imaging and glucose monitoring (see Section 3 for device-specific examples). These ML innovations are significantly enhancing disease management for clinicians and patients [45,46]. The adaptability of RNNs to diverse sequential inputs is particularly advantageous when time-dependent changes are critical for diagnosis and management [47,48].

While RNNs excel in time-dependent analysis, ensemble-based ML techniques, such as RFs and gradient boosting machines like XGBoost, are particularly useful in classification and regression tasks within POC analytics [49,50]. These classical ML techniques differ from most DL techniques by providing human-readable and explainable outputs. Explainability remains critical in ML-enabled healthcare, and tools such as SHAP (SHapley Additive exPlanations) help quantify feature contributions, aiding clinical understanding and fostering greater physician trust in ML-supported decisions [51]. Explainable ML models using RFs, XGBoost, and ANNs also appear to enhance clinical predictive accuracy, making them very useful for risk stratification and disease management applications where complex interactions exist and both patients and physicians require explainable rationale [51,52,53,54,55].

Transformer architectures, originally developed for natural language processing (NLP), are increasingly used in biomedical signal modeling. Transformers have attention mechanisms that allow the model to focus on relevant segments of complex, multimodal data. This ability to focus gives transformers a significant advantage over most other ML and DL models. Transformer models, including hybrid attention frameworks, enhance interpretability and prediction quality by isolating signal-frequency components [56,57]. Unfortunately, transformer models require significant computing resources, which are not often available on POC devices. Recently, model compression techniques, such as knowledge distillation, have been employed to enable transformer deployment in resource-limited POC devices [58]. Knowledge distillation is a technique where a smaller “student” model is trained to replicate a larger “teacher” model’s behavior, enabling efficient deployment while maintaining performance.

### 2.2. Real-Time Decision Support and Predictive Analytics

One of the most impactful domains for ML in POC devices is delivering real-time insights to healthcare providers and patients. Continuous processing of physiological signals enables ML models to generate alerts, predictions, and recommendations for timely intervention [59]. Continuous glucose monitoring (CGM) systems equipped with ML (primarily RNNs and long short-term memory [LSTM] networks coupled with RFs) can predict glucose excursions and suggest insulin adjustments, reducing risks of severe hypoglycemia or hyperglycemia [59]. Wearable or POC cardiac monitors employing real-time ECG data analysis (again using RNNs and/or CNNs) can promptly detect arrhythmic episodes or ischemic changes, triggering immediate notifications for patient or clinician action. One such example is KardiaMobile, a POC cardiac sensor system developed by AliveCor, which uses ML methods primarily based on CNNs for ECG signal classification [60,61]. It is able to detect arrhythmias, such as atrial fibrillation (AFib), bradycardia, and tachycardia. The system may also incorporate SVMs and RFs for specific rule-based classifications or postprocessing tasks. Such real-time decision support applications are vital for diagnosing medical conditions in which early recognition and treatment profoundly affect patient health or outcomes [60,61,62].

By incorporating ML analytics into smartphone applications and screens, POC device users can access risk alarms and obtain real-time insights and predictive analytics, improving both patient self-management and clinical decision-making [62]. Deploying ML on wearables requires balancing accuracy and computational efficiency. Studies show that lightweight models using knowledge distillation can achieve high accuracy while reducing memory and CPU requirements for POC applications [58]. Edge computing is another innovation that enables data processing on local hardware (instead of the cloud). This approach decreases latency and preserves privacy by minimizing cloud transmission. One example of an edge computing/knowledge distillation system can be found in the “light” transformer-based GlucoNet system for CGM, which uses these hardware innovations to combine feature decomposition with fast inference to predict blood glucose trends in real-time [58]. Another edge CGM system, namely, the Accu-Chek SmartGuide, incorporates RNN and LSTM-powered prediction engines to support proactive glycemic control [59,63].

Regardless of whether the implementation is on the POC itself or in the cloud, the integration of real-time ML-driven decision support and predictive analytics within clinical workflows has profound implications for POC-based healthcare. First, it enhances the accuracy and quality of clinical decisions by providing intuitive visualization tools and prioritized alert systems [43]. This greatly reduces the cognitive and service burden on both POC users and healthcare providers. Second, explainable ML algorithms further reduce alarm fatigue by providing context and confidence scores alongside alerts, thereby improving trust and actionable decision-making [51]. Third, these real-time ML tools foster increased patient engagement and self-management by empowering users with timely feedback, reducing hospital readmissions, and improving the overall health outcomes [32].

### 2.3. Personalization

Personalization is a core objective of AI-driven POC systems. This is because it both enables and encourages dynamic adaptation of treatment regimens to individual patient needs. For example, in diabetes management, many ML models continuously refine insulin dosing by learning from each patient’s unique glucose response, diet, and lifestyle. This personalization improves glycemic control, reduces complications, and enhances the quality of life [45]. Similarly, smart cardiac monitoring platforms can individualize alert thresholds and risk stratification criteria based on prior arrhythmic history and patient-specific signal features, minimizing false alarms and improving clinical relevance [7]. The incorporation of digital lifestyle factors, such as activity levels, stress, and sleep patterns, can further refine or enhance personalization [64]. Personalized AI models have been shown to increase patient adherence and satisfaction by aligning treatment with daily routines and preferences, thereby fostering sustainable disease self-management [65].

Adaptive learning algorithms, many of which incorporate RL, underpin personalization by tuning the model parameters in response to longitudinal patient data. For example, RL approaches have been deployed in closed-loop insulin delivery systems (“artificial pancreas”) to automatically optimize insulin delivery based on continuous sensor feedback [66]. Another example can be found with digital health biomarkers (DHBs) derived from multimodal wearables that include biochemical, physiological, and behavioral sensors. Use of these DHBs facilitates fine-grained patient profiling and enables more personalized recommendations. Examples of multimodal wearables that incorporate DHBs include GlucoTrack, which noninvasively monitors glucose using ultrasound, electromagnetic, and thermal sensing. Another is Biobeat, which tracks blood pressure and cardiac output; and yet another is the Dexcom G7, which combines CGM with behavioral data to personalize recommendations. Devices such as KardiaMobile integrate physiological and behavioral inputs for arrhythmia detection, whereas sweat-based biosensors such as the Gatorade Gx Patch assess hydration and stress to provide personalized, behavior-based feedback.

Recent innovations in smart wound care and infectious disease risk prediction have started to employ ML with multiple POC sensors to individualize monitoring and treatment plans and respond dynamically to changes in patient conditions. For instance, smart bandages equipped with POC sensors measure temperature, pH, and moisture, and ML models coupled to these sensors predict an individual’s likelihood of infection or delayed healing [67,68]. Examples include Tissue Analytics, WoundAide, and eKare insight. These systems use DL and computer vision to assess wound characteristics from images, while another smart wound care system, WoundVision Scout, uses thermal imaging and ML to detect pressure ulcers early. These wound care technologies are enabling better, data-driven clinical decisions and appear to be improving outcomes [67,68].

### 2.4. Automation and Workflow Optimization

AI and ML significantly reduce the need for manual data management and support workflow automation and optimization within clinical and home care environments. Automated data processing pipelines handle signal calibration, artifact filtering, quality control, and anomaly detection, minimizing the need for manual oversight. By reducing routine tasks and streamlining diagnostic workflows, ML integration decreases clinician workload, lowers human error rates, and improves overall system efficiency. Furthermore, ML models can assist in optimizing clinical pathways, triaging patients based on risk profiles, and prioritizing interventions, which is particularly valuable in resource-constrained settings. Automated signal processing pipelines found in KardiaMobile and ScanWatch (for AI-driven ECG analysis), Dexcom G7 and Accu-Chek SmartGuide (for glucose prediction), Tissue Analytics and WoundVision (for smart wound assessment), and Oura Ring and Empatica E4 (for sleep, stress, and seizure monitoring) include auto-calibration, artifact filtering, and internal quality control. Smart, AI-enabled automated signal processing ensures reliable sensor output with minimal user involvement. Decision support algorithms that automate the generation of alerts and dosage recommendations not only enhance efficiency but also reduce human errors [46]. By automating repetitive tasks with machines and ML, clinicians and patients are freed from routine manual monitoring, allowing them to focus on less tedious tasks. This capability impacts chronic disease management, where monitoring must be frequent and resources are often limited [69]. Beyond device automation, ML and AI facilitate optimization of clinical pathways by streamlining diagnostics in resource-constrained environments. One interesting way this has been achieved is through integration with the Internet of Medical Things (IoMT). This integration enables data aggregation from multiple POC and non-POC medical devices, supporting remote monitoring and telemedicine frameworks [70]. In infectious disease screening and emergency imaging, such as ultrasound, ML methods that use the IoMT help triage patients, prioritize interventions, optimize healthcare resources, and improve system responsiveness [28,33].

### 2.5. ML-Suitable POC Sensors and Devices

**Figure 4:**
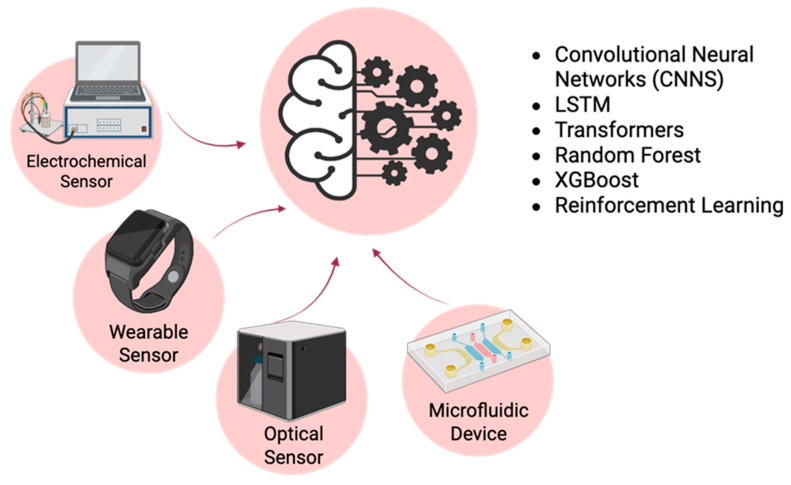
Key Areas of ML integration in POC devices to enhance diagnostics: data analysis and pattern recognition, real-time decision support, predictive analytics, personalization, automation, and workflow optimization. These integrations enable more accurate, timely, and personalized healthcare delivery through improved data interpretation, adaptive treatment planning, and operational efficiency.

Not all POC devices are adaptable to, or suitable for, AI and ML integration. The most suitable POC systems are typically electrochemical biosensors, optical sensors, wearable sensors, and microfluidic devices. These devices generate large amounts of data that are best analyzed by ML algorithms. Furthermore, they are ideally suited to provide ML-enabled insights into patient health (Figure 4).

#### 2.5.1. Electrochemical Sensors

Electrochemical sensors are fundamental to POC diagnostics because they offer excellent sensitivity, selectivity, and cost-effectiveness [33,71]. Their function revolves around measuring electrical signals such as current, voltage, or resistance produced by electrochemical reactions at the POC sensor surface [33]. These reactions are affected by the presence and concentration of the target analytes [72]. This electrical transduction mechanism allows for the facile detection of various biomarkers and analytes in biological fluids. The simplicity and potential for miniaturization of electrochemical sensors makes them ideal for diagnostic POC applications.

Various types of electrochemical sensors, including amperometric, potentiometric, and conductometric sensors, can be used to detect different biomarkers [72,73,74]. Amperometric sensors, which measure the current generated by analyte oxidation or reduction at a constant potential, exhibit high sensitivity and are often most suitable for glucose monitoring in diabetes management [73]. Common examples include continuous glucose meters, such as Accu-Chek and OneTouch Ultra, which use glucose oxidase or glucose dehydrogenase to generate an electrical current proportional to blood glucose levels. They also include lactate sensors, such as the Lactate Scout, which apply similar principles using lactate oxidase for real-time monitoring in sports activities and in critical care.

Potentiometric sensors measure the potential difference between electrodes without significant current flow and determine ion concentrations in bodily fluids [72]. Common examples of potentiometric POCs include ion-selective electrodes (ISEs) used in portable electrolyte analyzers to detect sodium, potassium, calcium, and chloride levels in the blood or urine. Other kinds of devices, such as the i-STAT bedside clinical testing system, incorporate potentiometric sensors for rapid bedside electrolyte testing.

Conductometric POC sensors measure changes in solution conductivity owing to the presence of certain medically relevant analytes [75]. The Abbott i-STAT system not only has potentiometric sensors but also conductometric sensors. Indeed, several i-STAT cartridges utilize conductometry for blood electrolyte and hematocrit measurements. Additionally, several nano-sized biosensors have been developed that detect urea or creatinine based on enzymatic reactions, aiding in kidney function monitoring [76]. Moreover, some electronic urine dipstick readers use conductometric principles to detect glucose or proteins by analyzing ionic shifts in urine samples [77].

Supervised ML algorithms require labeled datasets to train models that can predict either discrete categories (classification) or continuous values (regression). In classification, models learn from labeled data to assign inputs into predefined groups, for example, categorizing electrochemical biosensor outputs as “positive” or “negative” based on specific biomarkers. Common classification algorithms include RF, k-Nearest Neighbors (k-NNs), and Naïve Bayes. Regression, on the other hand, is used when the goal is to predict continuous numerical outputs, with widely used methods such as linear regression, logistical regression, XGBoost, and SVM. Beyond regression and classification, ML algorithms such as autoencoders, wavelet neural networks (WNNs), CNNs, RNNs, and LSTM can also be used to reduce noise, enhance signal-to-noise ratios, and improve sensitivity and specificity [72]. ML methods can also be used for prediction and predictive modeling. As noted earlier, ML models based on RNNs and LSTMs are frequently used to create personalized treatment recommendations based on readouts from electrochemical sensors. For instance, these systems can be used to predict the risk of hypoglycemic events in patients with diabetes based on CGM data from glucose sensors [59,63].

In contrast, unsupervised learning is increasingly being used to interpret complex, high-dimensional electrochemical signatures where labeled datasets are scarce. For instance, clustering and dimensionality-reduction methods such as principal component analysis (PCA) or t-distributed stochastic neighbor embedding (t-SNE) can reveal latent signal patterns and detect anomalous electrochemical responses [78]. These exploratory approaches enhance diagnostic insight when supervised labels are limited or unavailable.

The selection between supervised and unsupervised learning methods depends on the specific objectives and requirements of the POC. Key factors influencing this decision include the nature of the data (labeled vs. unlabeled), the goals of the predictive model, and the most appropriate algorithm for the POC data, considering the relevant features and attributes. As shown by a number of studies and by the existence of several commercial POC products, both supervised and unsupervised ML integration can significantly enhance the capabilities of electrochemical POC sensors, transforming them from crude measurement tools to sophisticated diagnostic systems [72,79].

#### 2.5.2. Optical Sensors

POC optics and sensors, including colorimetric, fluorometric, and surface plasmon resonance (SPR) sensors, are well-suited for ML integration, especially when used to detect clinically relevant analytes [80,81,82]. Colorimetric sensors rely on simple color changes to detect or quantify analytes and allow diagnostic decisions to be made. Colorimetric POC systems that incorporate ML are becoming increasingly common, owing to their simplicity and enhanced diagnostic accuracy. Smartphone-based colorimetric lateral flow assays (LFAs), such as those developed by mHealth platforms and Clip Health (formerly Luminostics), use smartphone images analyzed by ML algorithms, such as CNNs or SVMs, to quantify color intensity. These can be used to detect conditions such as COVID-19, HIV, or pregnancy [83,84,85]. Similarly, apps such as Dip.io and Healthy.io interpret urine dipstick color changes for assessing kidney function, glucose, protein, and infection screening using ML-based image processing. Paper-based microfluidic devices (μPADs) also employ ML to assess colorimetric changes related to analytes, such as glucose, creatinine, or heavy metals, especially in low-resource settings [86].

Fluorometric sensors that measure fluorescence emission offer high sensitivity and selectivity [82]. For example, smartphone-based fluorescence readers, such as those developed by Clip Health or Cue Health, utilize ML algorithms to interpret fluorescence signals from LFAs or microfluidic cartridges for detecting pathogens such as SARS-CoV-2, influenza, or sexually transmitted infections [83,84,85,87]. In cancer diagnostics, portable fluorescence readers equipped with ML are used to analyze fluorescence-tagged antibodies or fluorescent nucleic acid probes for on-site detection of circulating tumor markers [88].

SPR sensors that exploit refractive index changes near metal films are now being used to enable sensitive label-free detection [80]. These compact systems detect changes in refractive index upon target binding, allowing real-time monitoring of proteins, antibodies, or pathogens. SPR-based POC devices are increasingly used for infectious disease detection, cancer biomarker screening, and therapeutic monitoring [89,90].

In supervised learning, optical POC assays often employ classification algorithms such as CNNs to distinguish between positive and negative lateral flow test results or to classify infection types based on readouts from fluorescence spectra [83]. Regression techniques, such as partial least squares regression or gradient-boosted tree regression, can be used to estimate analyte concentrations by examining subtle variations in colorimetric or SPR signal intensity [81,89].

Unsupervised methods have also demonstrated their value in optical sensing, particularly for spectral data analysis. Techniques such as PCA or clustering can separate overlapping fluorescence spectra, reduce background noise, and uncover hidden patterns in complex multiplexed optical assays [91]. These approaches enhance sensitivity and facilitate the discovery of unexpected biomarker relationships.

Advances in microfluidics, miniaturization, ML, and interface design have enabled SPR platforms to move from lab settings to clinical and field applications. Overall, the data from SPR sensors or other optical sensors, whether as images or spectral information, are well suited for ML analysis [82].

#### 2.5.3. Imaging Systems

POC imaging systems are transforming medical diagnostics by enabling portable, real-time visualization in both clinical and remote settings. Examples include handheld ultrasound devices, such as the Butterfly iQ+ and Philips Lumify, which connect to smartphones for POC cardiac, abdominal, and obstetric assessments. Portable X-ray systems such as MinXray and Nano X are used in trauma care and mobile clinics, whereas smartphone-based otoscopes and dermatoscopes such as CellScope and DermLite allow high-resolution imaging of ears and skin. These systems are often supported by AI software for diagnostic assistance. POC microscopy platforms include low-cost solutions such as the MobileODT EVA System for cervical cancer screening and D3 systems that combine fluorescence and smartphone imaging for infectious disease detection. Additionally, AI-enhanced apps, such as Peek Vision, facilitate POC eye examinations and retinal assessments in underserved regions. These imaging systems are often integrated with ML to improve access, accuracy, and speed. For instance, ML algorithms can be used to enhance image quality, automate analysis, improve quantification, and perform pattern recognition. CNNs are particularly suitable for image analysis and are used by Butterfly iQ and Philips Lumify to assist with ultrasound image acquisition, real-time image enhancement, artifact reduction, and automated anatomical labeling. CNNs are also used in the MobileODT EVA System for cancer diagnosis and the D3 Systems for infectious disease detection [92,93]. CNNs have also been applied to analyze microfluidic chip images of blood samples for automated red and white blood cell counting [94,95].

#### 2.5.4. Microfluidic Platforms

Microfluidic devices are increasingly being used to integrate multiple sensor technologies and to automate complex diagnostic workflows [96]. Their capacity to miniaturize procedures, reduce sample volumes, and enhance portability makes them ideal for POC applications. Microfluidic platforms such as Sight Diagnostics’ OLO and Abionic’s abioSCOPE use microfluidics for rapid blood analysis and apply ML algorithms to interpret cellular or molecular patterns. Similarly, systems such as Cue Health and Lucira rely on microfluidic cartridges combined with ML-driven signal processing to detect viral RNA or antigens. Cepheid’s GeneXpert integrates microfluidics with ML-enhanced PCR data interpretation for rapid infectious disease diagnosis. Many microfluidic devices can integrate multiple sensor types, such as electrochemical, optical, and piezoelectric sensors, whose multiparametric outputs, along with image and flow data, are ideally suited for ML analysis [72,91]. CNNs are widely used for interpreting colorimetric assays and identifying cells or particles in microfluidic images, whereas SVMs, RFs, and gradient boosting methods are best used for classifying biological samples and detecting assay types [72]. RNNs and Gaussian processes are used in soft sensing to estimate hidden variables, and deep neural networks (DNNs) can infer fluid properties from flow patterns. Overall, the incorporation of ML significantly enhanced microfluidic platforms, paving the way for more advanced and intelligent diagnostic systems [17,72,91].

#### 2.5.5. Wearable Sensors

Wearable sensors, such as smartwatches (Apple and Samsung Galaxy Watches) and fitness trackers (Fitbit, Google Pixel Watches, and Amazfit), are revolutionizing health monitoring by enabling the continuous and non-invasive collection of physiological data [74,97,98]. These wearable sensors can measure a range of biological signals, including bioelectrical signals such as ECGs, EEGs, EMGs, and PPGs, as well as biochemical parameters such as glucose levels or electrolytes, and physiological data, including body temperature, heart rate, blood pressure, and motion or activity data [7,10,26,74,97,99]. Continuous monitoring facilitates the early detection of health issues, supports personalized medicine, and promotes proactive health management. Since wearable devices generate large, continuous time-series datasets, this makes them particularly suitable for supervised learning approaches. Classification ML models (e.g., CNNs and SVMs) can distinguish between arrhythmic and normal ECG segments or classify physical activity states from accelerometer data [28,97,100,101]. Regression approaches, such as LSTMs, are commonly used to predict physiological parameters, including future glucose levels or heart rate variability [48,49,74]. Wearable ECG monitors (e.g., Apple Watch and KardiaMobile) employ CNN classifiers to distinguish between normal sinus rhythm and arrhythmias such as atrial fibrillation, bradycardia, or tachycardia [61].

By leveraging the “big” data, ML algorithms can detect patterns and anomalies, predict future health events, and deliver personalized feedback to users. The most effective ML methods include CNNs for processing time-series and multichannel sensor data (e.g., ECGs or PPGs), RNNs or LSTMs for capturing temporal dynamics, and RFs or SVMs for classification tasks, such as activity or stress detection [28,72]. At the same time, unsupervised learning is valuable for wearables because labeled training data are often sparse. Clustering and anomaly-detection models can flag abnormal heart rate or activity patterns without prior labels, enabling early warning for conditions such as infections, sleep disorders, or heart failure decompensation [102,103,104]. These unsupervised techniques thus play a crucial role in continuous health monitoring where unexpected physiological changes must be identified. Autoencoders and unsupervised clustering methods are particularly useful for anomaly detection and pattern discovery in continuous physiological monitoring [55,101]. For instance, RNNs, CNNs, and SVMs have been used to identify arrhythmias from ECG data [48,97] or forecast hypoglycemic episodes using continuous glucose-monitoring data [55,74]. The integration of ML with wearables holds significant promise in enhancing healthcare interventions and improving patient outcomes.

## 3. Detailed Examples of POC Devices Using ML

### 3.1. ML-Powered Continuous Glucose Monitors (CGMs)

Continuous Glucose Monitors (CGMs) have transformed diabetes management over the past 10–15 years [45,59,65,105,106,107]. These devices provide users with continuous, real-time glucose readings, typically updated every one to five minutes, thereby delivering a detailed glycemic profile that surpasses the quality of traditional, manual, episodic self-monitoring of blood glucose (SMBG) methods. Unlike SMBG, which relies on discrete fingertip measurements that may miss transient but critical hypo- or hyperglycemic events, CGMs offer a dynamic picture of glucose fluctuations throughout the day and night. This real-time data significantly improves patients’ ability to manage exogenous insulin dosing and anticipate forthcoming adverse glycemic events by tracking both current blood glucose concentrations and their rate of change in near real-time [45]. The utility of CGMs is highlighted in the management of type 1 diabetes (T1D), a condition that requires meticulous regulation of insulin. Continuous data from CGMs enable more accurate insulin dosing decisions, potentially reducing both hypoglycemia and hyperglycemia risk. Moreover, CGMs support clinical decision-making by allowing both patients and healthcare providers to analyze glucose patterns and tailor therapies accordingly [106]. This real-time feedback loop advances beyond glucose measurement toward predictive and preventive diabetes care. Owing to technological advances, CGMs now interface seamlessly with mobile phones and insulin pumps, providing a more integrated and responsive approach for glycemic control [65].

The integration of machine learning (ML) methodologies into CGM systems has markedly enhanced their efficacy by enabling predictive analytics that anticipate glucose trends and optimize insulin delivery. As discussed in Section 2, ML algorithms, ranging from traditional ensemble methods to advanced deep learning (DL), are now widely employed in many CGMs for forecasting glucose levels, predicting hypoglycemia, and facilitating real-time decision-making [49,59,108,109]. These models assimilate CGM data along with patient-specific physiological and behavioral inputs (e.g., meal intake, physical activity, and sleep patterns) to generate personalized glycemic forecasts and provide timely insulin bolus recommendations. A recent hybrid framework shows the feasibility of deploying efficient glucose prediction models using lightweight transformers for CGM signal interpretation [49]. It couples a time series-based ML model with XGBoost and achieves sub-10 mg dL^−1^ RMSE for 15 to 60 min prediction horizons while relying solely on CGM signals. This advancement underscores the clinical potential of adaptive algorithm tuning, which continually learns from historical and incoming data to refine insulin dosing advice and reduce adverse glycemic excursions. Moreover, state-of-the-art ML-powered CGM platforms have increasingly exploited edge computing paradigms. Techniques, such as knowledge distillation, have also been applied to compress complex ML models (esp. transformers), allowing their deployment on resource-constrained edge devices with minimal latency while maintaining high predictive accuracy [66].

Despite these advances, challenges still persist related to sensor data variability, noise artifacts, and inter-individual differences. More robust data preprocessing, better calibration strategies, and improved algorithmic resilience are required to ensure better performance [46].

Examples of commercially available CGM systems integrating advanced ML capabilities include FreeStyle Libre 3, Dexcom G7, and Medtronic Simplera. These devices exemplify the fusion of advanced sensor technology with ML-enabled predictive analytics for diabetes care. In a 2024–25 interventional study of adults with type 1 diabetes, Eichenlaub et al. found that FreeStyle Libre 3, Dexcom G7, and Medtronic Simplera had broadly comparable mean absolute relative differences (MARDs) when compared to venous YSI (11.6%, 12.0%, and 11.6%, respectively [110]). However, accuracy diverged depending on the comparator method, with FreeStyle Libre 3 and Dexcom G7 generally showing stronger performance than Medtronic Simplera against capillary and venous comparators, whereas Medtronic Simplera performed better in hypoglycemia [110]. Building on those findings, Freckmann et al. analyzed 14-day, parallel-wear data from 23 participants and showed that discordant glucose profiles led to clinically significant differences in therapy metrics [111]. Libre 3 and Dexcom G7 tended to agree more closely, while Medtronic Simplera systematically reported lower glucose values, leading to higher time in range (84% vs. ~76%) but also higher time below range. Within individuals, differences in time in range (TIR) exceeded 5% in 22% of Libre 3 vs. Dexcom G7 comparisons, 74% of Libre 3 vs. Medtronic Simplera, and 52% of Dexcom G7 vs. Medtronic Simplera, with glucose management indicator (GMI) values differing by more than 0.3% in a substantial fraction of participants [111]. These discrepancies mean that treatment decisions, including whether glycemic targets are achieved, may depend strongly on which CGM system is used.

The Dexcom G7 received FDA clearance in December 2022 as an integrated CGM (iCGM) under 21 CFR 862.1355, with an expanded 15-day wear option cleared in April 2025 [112]. Medtronic’s Simplera was CE-marked in Europe in 2023, FDA-approved in August 2024, and its Simplera Sync configuration was approved in April 2025 for use with the MiniMed 780G AID system [113].

Data transmission from these CGM sensors typically employs Bluetooth low energy (BLE) protocols to stream blood glucose readings to connected smartphones, where more CPU- and power-hungry, ML-driven applications provide dynamic glucose trend visualizations and predictions. For instance, Accu-Chek (AC) SmartGuide integrates a glucose prediction suite powered by ML into its smartphone user interface, enabling proactive glucose management for people with diabetes through predictive alerts and recommended therapy adjustments [59]. Clinical evidence suggests that these ML-augmented CGM systems improve therapeutic decision-making, leading to better glycemic control and reducing the frequency and severity of hypo- and hyperglycemic events [114].

### 3.2. Portable Imaging Devices

Portable imaging devices such as portable ultrasound, portable MRI, portable X-rays, and handheld optical microscopes have revolutionized point-of-care (POC) diagnostics by enabling real-time imaging in diverse clinical environments, including remote and resource-limited settings. These handheld imaging devices combine low-cost hardware, wireless connectivity, portability, and ease of use to facilitate rapid diagnostic assessments in or near patients. Portable imaging systems help to bridge the gap between advanced imaging modalities and frontline care. The benefits include reduced examination times, immediate clinical decision support, and the ability to reach underserved populations without direct access to larger imaging centers [115]. However, traditional ultrasound interpretation often requires specialized expertise. Furthermore, inter-operator variability can limit diagnostic consistency. This challenge is especially pronounced in low- and middle-income countries (LMICs), where ultrasound expertise is scarce. Consequently, there is a critical need for automated image interpretation systems to mitigate these problems, thereby enhancing diagnostic accuracy and expanding the utility of portable ultrasound [32].

ML techniques, particularly DL models, deeply involved in tasks associated with image recognition analysis, have been instrumental in transforming ultrasound image processing. These models can be used to perform segmentation of anatomical structures, extraction of quantitative imaging features, and classification of pathological abnormalities with high precision. Ultrasound ML image processing pipelines often include preprocessing steps, such as noise filtering and artifact reduction, to enhance image quality, followed by feature classification, culminating in automated diagnostic suggestions or decision support [60, 116]. One key advantage of ML integration in ultrasound imaging is the facilitation of real-time anomaly detection, which can assist clinicians or non-expert operators in identifying conditions, such as cardiac dysfunction, lung pathologies, or focal lesions. This can be achieved by training the CNN models on expertly annotated ultrasound image datasets, enabling these algorithms to detect subtle imaging features that may elude manual interpretation. In addition, explainable ML technologies are being developed to provide interpretative insights that bolster clinician trust and adoption. Regulatory frameworks are evolving to ensure the safety and efficacy of ML-powered ultrasound imaging systems through rigorous clinical validation and adherence to software-as-medical-device (SaMD) standards [117].

The Butterfly iQ is a popular handheld ultrasound device with ML capabilities, specifically designed for POC use. This device is connected to smartphones and tablets, leveraging their processing power and display interfaces to deliver versatile imaging across multiple clinical applications. As previously mentioned, the Butterfly iQ incorporates DL algorithms that enable automated image interpretation for cardiac and pulmonary assessments. These ML tools provide real-time guidance and preliminary diagnostic information to users irrespective of prior ultrasound training. They achieve excellent performance metrics, such as an area under the curve (AUC) of >0.90 for lung conditions and a mean absolute error (MAE) of <5% for cardiac assessments, even on low-power platforms [4,32]. Since 2023, clinical validation of Butterfly iQ handheld ultrasound has expanded across emergency and specialty care. In a randomized clinical trial involving 110 emergency department patients, the Butterfly iQ demonstrated diagnostic accuracy on par with a cart-based system for cardiac, lung, renal, aortic, and biliary scans, achieving a sensitivity of 92.9%, a specificity of 92.3%, and an overall accuracy of 92.5%, thereby establishing its noninferiority for common POC indications [118].

Similarly, ophthalmological data has shown strong agreement: in retinal detachment triage, the Butterfly iQ+ achieved 90% sensitivity and 95% specificity with κ = 0.85 against clinical and surgical diagnoses. This matches the performance of a conventional ophthalmic B-scan [119]. Prehospital research also supports the utility of handheld devices. For example, thoracic ultrasound with Butterfly improved the diagnosis of acute heart failure and reduced the time to treatment initiation, underscoring the value of portable devices in field care [120].

Regulatory progress for POC ultrasound has been swift, with FDA 510(k) clearance of the Butterfly iQ3 on 4 January 2024 [121], along with expanded AI functionality following the clearance of the Auto B-Line Counter for lung ultrasound (Butterfly Network) [122]. Other POC ultrasound applications appear to be on the horizon. Performance studies in ultrasound bladder volume assessment suggest convenience advantages for Butterfly’s auto volume tool, although conventional cart ultrasound still shows the highest agreement with catheterization [123]. These clinical studies have demonstrated the effectiveness of the Butterfly iQ in various settings, ranging from emergency departments to rural clinics, affirming its potential to democratize advanced ultrasound imaging technologies and enhance diagnostic reach globally.

### 3.3. Wearable Cardiac Monitors

Wearable technologies, including smartwatches and fitness trackers (such as Samsung Galaxy Watches [Series 4 and later], Apple Watches [Series 4 and later], and Fitbit [Sense and Sense 2]), have emerged as transformative tools for continuous cardiac surveillance. These devices, equipped with PPG and single-lead ECG sensors, enable the long-term collection of heart rate and heart rhythm data, thereby facilitating the early detection of arrhythmias, such as AFib and other cardiac anomalies. Their accessibility fosters patient engagement in cardiovascular self-monitoring while allowing clinicians to remotely assess at-risk individuals. This enables earlier interventions and improves the outcomes. In one study, an ML model trained on multisensor data from wearable devices predicted heart failure decompensation events with 63% sensitivity and 92% specificity [124]. Several challenges remain, such as balancing sensor precision with user comfort, handling continuous data streams, and ensuring data privacy and security. False-positive arrhythmia alerts can also raise patient anxiety and lead to unnecessary healthcare use. Thus, developing advanced ML algorithms with high specificity and sensitivity is essential for clinical reliability [125].

ML methodologies employed for arrhythmia detection with wearables commonly involve supervised learning frameworks capable of recognizing subtle and complex patterns within noisy real-world signals. CNNs are frequently utilized because of their capacity for hierarchical feature extraction from ECG waveforms, whereas RNNs, including LSTM models, are advantageous for modeling temporal dependencies within sequential data. Increasingly, explainable ML approaches are being integrated into healthcare to improve the transparency and trustworthiness of model outputs among clinical users [100]. In parallel, cloud-based infrastructure and IoMT frameworks have facilitated real-time remote monitoring and decision support, further advancing personalized approaches to cardiac care [62].

The Apple Watch (Series 4 and higher) is one of the most popular consumer-grade wearables that integrates ML-driven cardiodiagnostic capabilities. Equipped with both PPG and ECG functionalities, it facilitates intermittent ECG acquisition along with continuous heart rhythm monitoring. Embedded ML algorithms analyze incoming data to identify AFib and other rhythm disturbances and provide real-time alerts to users. The Apple Heart Study reported a positive predictive value (PPV) of 84% for AFib detection alerts generated by the Apple Watch [126]. This result underscores the potential of smartwatch-based screening for ambulatory populations. In a subsequent clinical validation study involving postoperative patients, newer-generation Apple Watches demonstrated a sensitivity of 73.9% and specificity of 95.7% for AFib detection when compared to gold-standard telemetry [98]. This result highlights the potential for improved accuracy with the inclusion of on-device, single-lead ECG recordings [62]. Additionally, the integration of this output with personalized health applications fosters patient engagement, education, and adherence to lifestyle modifications.

Independent validation studies indicate that the single-lead ECG application achieves an overall sensitivity and specificity of approximately 95% for detecting atrial fibrillation [127]. Apple’s AFib History feature, FDA-cleared in 2022 and formally qualified in 2024, provides accurate weekly AF burden estimates within ±5% of reference patches [128]. In 2024, the Sleep Apnea Notification received 510(k) clearance, with ~66% sensitivity and >95% specificity against polysomnography [129]. Nevertheless, questions regarding the long-term clinical impact of these wearable systems remain active areas of investigation [7,125].

### 3.4. ML-Enhanced Infectious Disease Detection

Rapid and accurate POC detection of infectious diseases is vital to control outbreaks and initiate timely treatment. ML-based approaches have significantly enhanced the diagnostic accuracy of infectious diseases by improving test result interpretation and reducing false-positive and false-negative diagnoses in diseases such as malaria and COVID-19. ML systems analyze complex patterns in biochemical signals, imaging data, or combined sensor outputs to support decision-making in environments where laboratory infrastructure may be limited. This fusion of ML with POC technologies is particularly impactful in LMICs and rural settings, where resource constraints necessitate efficient and accurate diagnosis [33].

The COVID-19 pandemic underscores the importance of continuous physiological monitoring for early disease detection. Employing wearable sensors that track vital signs, including heart rate variability and body temperature, ML algorithms can detect subtle, pre-symptomatic physiological alterations that are indicative of infection. The work conducted by Michael Snyder’s group at Stanford exemplifies this paradigm, with wearable sensor arrays integrated with ML analytics, enabling early infection identification (COVID-19 and Lyme disease) and continuous health surveillance [11,12,130]. Such approaches offer the potential to augment public health responses through real-time monitoring and outbreak predictions [131].

One prominent commercial example of a POC infectious disease detection system is the Cepheid GeneXpert system, which combines molecular diagnostics with ML-assisted analysis to provide rapid near-patient identification of infectious pathogens. This system is a rapid, fully automated, PCR-based diagnostic platform that integrates sample preparation, nucleic acid extraction, amplification, and real-time detection into a single cartridge, providing results within 30–90 min. It is widely used for detecting infectious agents, such as *Mycobacterium tuberculosis*, COVID-19, MRSA, *C. difficile*, HIV, and STIs. Supervised ML methods as discussed earlier are employed in the post-analytic stages for quality control, amplification curve classification, error detection, and predictive maintenance, illustrating how advanced ML can augment POC molecular testing, delivering actionable insights swiftly in a user-friendly format [32].

### 3.5. Smart Wound Care Systems

Chronic wounds impose substantial morbidity and healthcare costs, necessitating continuous monitoring to detect infections early and to optimize healing trajectories. Smart wound care sensors capture parameters, such as moisture levels, temperature, pH, and bacterial load. They can provide objective and continuous assessments that surpass the limited scope of manual inspection during clinical visits. Wound care sensors also support outpatient or home-based care models by delivering timely data to clinicians and patients alike, facilitating earlier interventions and reducing hospitalization.

ML models are increasingly being employed to analyze multidimensional data obtained from wound sensors. By applying ML-based classification algorithms and predictive analytics, these systems can identify patterns associated with the risk of infection, delayed healing, or other complications. This involves preprocessing sensor signals, selecting relevant features, and training models to classify wound status or forecast healing outcomes. The integration of these models into clinical decision support tools enables personalized evidence-based wound management protocols [69].

Swift Medical has developed a commercial ML-powered wound imaging and assessment platform (called Swift Skin) that employs computer vision and ML techniques to objectively analyze wound images and sensor data [132]. The system has shown very good diagnostic performance, with image segmentation models achieving Dice coefficients of >0.85 and classification tools reporting F1-scores of approximately 0.88. These data underscore the accuracy of the system in detecting infection risk and healing progress.

Swift Skin has shown robust clinical validation results in recent studies. A time-motion study comparing manual methods with Swift revealed that clinicians completed wound assessments nearly 79% faster using the application, with the average time for image capture and measurement being about half of what is required manually. Furthermore, Swift enhanced first-attempt success rates for high-quality wound images (92.2% vs. 75.7%) and significantly reduced workflow inefficiencies across various wound types, including diabetic, venous, surgical, and pressure ulcers [132].

The Swift Skin software utilizes an FDA-registered fiducial marker (HealX) to standardize color, size, and depth calibration, ensuring regulatory compliance for clinical use in the United States. Updated performance metrics confirm that the system not only saves 1–2 min per assessment but also improves image quality, reliability, and documentation accuracy, thereby enhancing clinician capacity and potentially affecting reimbursement outcomes [132]. Swift Skin offers clinicians detailed insights into wound size, tissue composition, and healing progression with validated improvements in diagnostic accuracy and early complication detection. By reducing reliance on subjective assessments, Swift Skin appears to be contributing to enhanced patient outcomes and lower healthcare utilization, making it a useful addition to chronic wound care [132,133].

### 3.6. ML Algorithms for POC Systems

Table 1 presents a comprehensive overview of the ML algorithms currently utilized in POC systems, highlighting their specific applications, strengths, and limitations. CNNs are widely used in image-based sensing tasks, such as handheld ultrasound and optical glucose spectroscopy, as well as in ECG and PPG signal-based arrhythmia detection, owing to their strong spatial feature extraction capabilities. As mentioned previously in Section 2, specialized time-series ML models are utilized for sequential physiological data, such as predicting CGM trends and examining ECG streams over time. Transformer-based models and hybrid attention mechanisms have been introduced for multimodal signal analysis, particularly for integrating data from sources such as CGMs, activity monitors, sleep tracking, and real-time interpretation of ultrasound data. For structured clinical data, RF algorithms are utilized in risk stratification dashboards and glucose level predictions, whereas XGBoost and other gradient-boosted tree models are preferred for trend prediction and real-time vital sign monitoring on portable devices. RL is emerging in adaptive therapeutic systems, particularly in closed-loop insulin delivery (also known as artificial pancreas systems), where the algorithm learns the optimal dosing strategies based on user feedback. Explainable AI techniques, such as SHAP, are increasingly being used to interpret model outputs post hoc, especially in CGM and ECG applications, supporting clinical decision-making by providing transparency. Finally, unsupervised learning and clustering approaches are being employed for patient stratification and anomaly detection, particularly when labeled data are scarce or unavailable. This enables the discovery of hidden patterns in wearable data streams.

**Figure 5:**
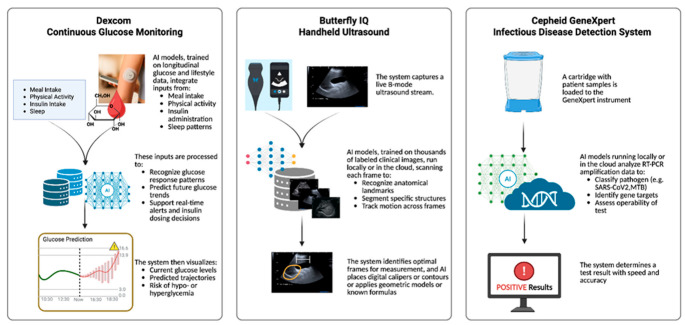
Examples of AI-enabled POC diagnostic and monitoring devices. (**Left**) Dexcom Continuous Glucose Monitoring integrates lifestyle and insulin data to predict glucose trends and support dosing decisions. (**Middle**) Butterfly iQ handheld ultrasound uses AI to recognize anatomical landmarks, segment structures, and assist with measurements. (**Right**) Cepheid GeneXpert infectious disease detection system leverages AI for RT-PCR data analysis, enabling rapid and accurate pathogen detection.

Figure 5 highlights three representative examples of AI-enabled commercial POC diagnostic and monitoring devices. The Dexcom CGM system combines glucose readings with contextual lifestyle data, such as meal intake, physical activity, insulin dosing, and sleep, to predict future glucose trends and guide insulin management. The Butterfly iQ handheld ultrasound uses DL models trained on thousands of clinical images to automatically recognize anatomical landmarks, segment structures, and provide real-time measurement assistance, making ultrasound more accessible to non-specialists. The Cepheid GeneXpert infectious disease detection system integrates cartridge-based RT-PCR testing with AI-based analysis to classify pathogens, identify gene targets, and deliver rapid, accurate infectious disease diagnoses. Collectively, these devices illustrate how AI and ML enhance POC platforms by improving diagnostic accuracy, supporting real-time decision-making, and enabling personalized healthcare.

Table 2 summarizes AI/ML-powered POC devices that are commercially available with application area, type of algorithm, clinical validation status, and features in comparison.

## 4. Gaps and Challenges in POC and ML Integration

### 4.1. Data Quality, Reliability, and Interoperability

While machine learning (ML) integration in point-of-care (POC) diagnostics holds transformative potential, several challenges are impeding its broad adoption in healthcare settings. Data variability, instrument noise, motion artifacts, and environmental interference can pose challenges to ML integration. Additionally, patient-specific signals complicate generalized pattern recognition. These issues require more sophisticated data preprocessing, such as filtering, normalization, and augmentation. Overfitting is addressed through regularization and cross-validation. Occasional POC device disconnections may lead to missing data, but ML-based imputation and gap-tolerant models can manage these gaps. Overall, advancements in dual-modal sensing and signal calibration and improving data input quality. This facilitates more accurate and reliable ML-driven diagnostics through robust data management [69,134].

ML-powered POC systems, especially those utilizing wearable and portable sensors, often face reduced accuracy outside controlled laboratory environments. Factors such as environmental conditions, physiological variability, sensor calibration drift, and signal artifacts can degrade data quality, thereby compromising model predictions. These limitations are particularly pronounced in applications requiring fine-grained detection, such as metabolic regulation, arrhythmia classification, and mental health monitoring, where even minor signal deviations hold clinical significance [45,46,48,101,103,134].

Data and model limitations can also hinder the reliability of the ML tools. Many ML models are trained on small homogeneous datasets, limiting their generalizability to more diverse or underserved populations [32,46,117,134]. Issues such as missing data, irregular sampling, and sensor noise further reduce model robustness. Mazgouti et al. [49] addressed this by applying a Savitzky-Golay filter to continuous glucose monitoring (CGM) data, improving the glucose prediction accuracy. This highlights the importance of signal preprocessing.

### 4.2. Technical Workflow Integration

Another barrier to adoption stems from problems with usability and workflow integration. Obtaining clinically relevant, high-quality data is not trivial—particularly in resource-constrained environments where preprocessing, calibration, and quality assurance pipelines may be incomplete or inconsistent. Beyond data capture, reliable connectivity is required to support cloud-based analytics, remote monitoring, and software updates, yet many low-resource or rural settings face unstable network infrastructure.

Although ML models can outperform manual interpretation in many diagnostic tasks (e.g., CNNs in medical imaging or RNNs in glucose forecasting), their “black-box” nature often undermines clinical trust. Explainability tools such as SHAP offer some transparency, but integration into clinical workflows is still inconsistent due to interface complexity and limited provider training [51,52,53,135]. Many clinicians also lack the digital literacy or institutional support needed to implement ML-generated recommendations effectively in time-sensitive environments [136].

Specific sensors and device-related issues also persist. Electrochemical sensors are prone to signal interference from surface fouling or sample contamination [72,74], and optical sensors require consistent lighting and image quality for reliable performance, even when enhanced by ML use [81]. Microfluidic devices face miniaturization and integration hurdles, particularly when embedding ML components in compact systems [17,117,137]. For wearable sensors, key challenges include battery life, computational constraints, and ensuring algorithm robustness across real-world conditions and diverse populations [74,97].

### 4.3. Ethical, Regulatory, and Bias Considerations

The use of ML in POC settings is beginning to raise ethical and regulatory concerns, including data privacy and accountability. Algorithmic bias is a particular concern, as models trained on small or homogeneous datasets may underperform in populations that differ by gender, ethnicity, age, or socioeconomic status. Such disparities can exacerbate existing health inequities if not carefully mitigated through the use of diverse training datasets, fairness audits, and transparent reporting practices.

Real-time monitoring of sensitive data such as heart rate variability, glucose levels, or emotional states in mental health introduces privacy risks, particularly when used outside traditional clinical settings [138]. This underscores the need for robust encryption, privacy-preserving AI methods, and transparent data governance frameworks. Informed consent becomes more complex in AI-enabled systems, as patients must understand not only how their data are collected and shared but also how algorithmic recommendations are generated and used in clinical decision-making [117,138]. Successful deployment of ML-driven POC devices must navigate data privacy regulations, ensure interoperability with electronic health records (EHRs), and comply with evolving regulatory frameworks such as the FDA’s Software as a Medical Device (SaMD) model and the proposed Predetermined Change Control Plan [117,136,138]. Privacy-preserving AI approaches are essential for maintaining patient trust, and adoption is further influenced by usability, clinician training, and the safety of ML-guided recommendations [136]. Sustained performance over time also requires multicenter validation and continuous model learning [111,117]. Incentivizing reimbursement and establishing robust validation standards are key enablers of widespread AI adoption. User training and change management will require close collaboration between developers, clinicians, and healthcare administrators. Continued efforts to develop interoperability standards and clinical guidelines are also critical to support effective ML-enabled workflows [117,138].

Biases in training data can lead to inequitable model performance across genders, ethnicities, or socioeconomic groups. These issues necessitate fairness audits and requirements for more transparent reporting [125,138]. Legal frameworks regarding liability in ML-assisted medical decisions remain underdeveloped, impeding broader clinical adoption [117].

### 4.4. Scalability and Access

Scalability and access to AI-powered POCs remain major challenges in low-resource and rural settings. One of the key selling points for AI-powered POC devices is their potential to alleviate disparities in healthcare access, particularly in rural, remote, and low- and middle-income countries (LMICs), where conventional laboratory infrastructure and specialist expertise are scarce. Advanced ML-integrated POC systems democratize diagnostics by reducing dependence on centralized facilities, thereby enabling earlier interventions and reducing preventable morbidity and mortality. However, barriers remain, including device cost, reliance on stable power supplies and internet connectivity, cultural adaptation of digital health tools, and long-term sustainability of deployment in low-resource settings [32,59,131]. These limitations restrict equitable access to high-quality diagnostics, especially in decentralized trials and community-based care [139]. Although edge deployment and model compression can reduce costs and latency [49,64,70], broader infrastructure investment is required.

Table 3 outlines some of the primary obstacles to effectively integrating AI into POC technologies, categorizing the challenges into six domains: technical limitations, data quality and generalizability, ethical and regulatory considerations, usability and workflow compatibility, scalability in low-resource settings, and sensor/device-related issues. Addressing these issues through improved preprocessing, diverse training datasets, explainability frameworks, and hardware optimization is essential for safe, equitable, and scalable deployment of ML-enhanced POC diagnostics.

## 5. New Directions for POC and ML Integration

### 5.1. Mental Health Monitoring

Mental health disorders, including depression and anxiety, represent a significant and escalating global health burden. The World Health Organization estimates that over 300 million people worldwide suffer from depression, and anxiety disorders are pervasive [141]. Despite this prevalence, mental health conditions frequently remain underdiagnosed and undertreated owing to stigma, resource limitations, and the subjective nature of traditional assessments. Early detection and continuous monitoring are critical, as they facilitate timely interventions that can mitigate disease progression and improve the quality of life [142]. Point-of-care (POC) devices offer promise in addressing this diagnostic gap by providing continuous, objective assessment tools capable of detecting subtle physiological and behavioral indicators of mental health [142,143]. They enable real-time tracking outside clinical settings and facilitate personalized feedback, thus overcoming barriers to care associated with typical episodic psychiatric evaluations.

Machine learning (ML) methodologies have rapidly evolved to exploit multimodal input data for mental health assessments through POC devices. For instance, spoken speech patterns are a particularly rich behavioral signal. Variations in tone, pitch, pace, jitter, shimmer, and pause duration mirror the underlying emotional states [144,145]. NLP techniques, sentiment analysis, semantic modeling, and vocal-biomarker extraction can reliably flag depressive or anxious content in real-time speech transcripts [146]. In parallel, computer-vision systems can analyze facial micro-expressions to detect emotional states or cues. CNNs capture subtle muscular movements, while RNNs or attention-based transformers model their temporal dynamics [147]. Multimodal fusion architectures that blend audio and visual streams enhance robustness against background noise, lighting variation, and cultural expression differences, enabling unobtrusive continuous monitoring in natural settings [148].

Emerging prototypes point toward POC devices that couple advanced multimodal representation learning with real-time language generation to create a fully closed therapeutic loop [149,150]. In this architecture, generative adversarial networks (GANs) are trained jointly on speech, facial micro-expressions, and physiological waveforms, yielding a latent manifold that preserves fine-grained patterns required for high sensitivity emotion recognition. To further enhance the capabilities of this system, fine-tuned large language models (LLMs) are proposed to sit atop the detector stack [151]. When the multimodal classifier infers a user’s affective state, the LLM adjusts the tone, formality, and emotional framing of micro-interventions (e.g., motivational prompts, paced-breathing cues, or CBT exercises), transforming passive sensing into just-in-time digital therapy.

Beyond behavioral cues, next-generation mental health monitoring POC units will likely integrate biosensors that stream heart rate variability, galvanic skin response, electrodermal activity, and salivary cortisol [143,152,153]. Temporal DL models (e.g., Bayesian LSTMs and probabilistic autoencoders) can learn personal baselines and surface deviations preceding depressive or anxiety episodes [154]. When physiological embeddings project onto a GAN-derived latent space and are interpreted by an LLM, the system could generate risk forecasts, mental health digests, and clinician alerts [155,156]. Such pipelines, which fuse physiological, acoustic, and visual features, promise proactive, personalized mental health care where traditional services are scarce. These integrated AML platforms illustrate a frontier in digital psychiatry and medicine.

### 5.2. Nutrition and Metabolic Health

Precision nutrition (PN) is a personalized approach to dietary recommendations that considers an individual’s physiology, microbiome, metabolome, and lifestyle to optimize health and prevent disease. The landscape of pN has been rapidly transformed by the convergence of POC systems, wearable devices, and AI [105,157,158,159,160]. These technologies enable real-time monitoring of metabolic markers and translate complex biological data into PN and wellness strategies. Recent advances from Michael Snyder’s lab at Stanford and our work on PN have demonstrated how ML-enhanced POC systems have the potential to deliver individualized nutritional insights and interventions [105,161,162].

One of the most compelling examples of this innovation comes from Snyder’s group, which pioneered the use of CGMs and digital health tools to characterize personalized glycemic responses. Their research revealed that glycemic reactions to specific foods vary widely between individuals, even when controlling for macronutrient content. This work identified distinct “glucotypes”, or patterns of glucose fluctuation, which offer a refined lens to assess glucose regulation and dietary impact [163]. Snyder et al. integrated CGM data with mobile apps and wearable devices to deliver dynamic feedback, enabling users to make real-time dietary adjustments [161,164]. Furthermore, these studies established that insulin-resistant individuals exhibit decreased gut microbial stability, underscoring the importance of microbiome dynamics in shaping personalized dietary responses. By leveraging multi-omic data from genomics, metabolomics, proteomics, and microbiome analyses with digital health tools, Snyder’s research has advanced personalized nutrition and established a model for ML-driven nutritional strategies.

Our own work, led by Dorsa Yahya Rayat, builds on this paradigm through an N-of-1 study that explores how personalized monitoring can inform PN [162]. In this study, the participant cycled through four distinct dietary regimens: fast food, mediterranean, ketogenic, and a habitual diet, each for two weeks, separated by 1-week washout periods. Throughout the intervention, continuous physiological data were collected using wearable devices that tracked heart rate, blood pressure, skin temperature, and other metrics. In parallel, biological samples (blood, urine, and stool) were collected daily to enable deep multi-omic profiling, including metabolomics, proteomics, genomics, and microbiome analysis. Automated, cognitive, and psychological assessments were also self-administered to provide a holistic view of health outcomes. The results demonstrated that dietary changes induce rapid (within three days) and profound measurable shifts across molecular, physiological, and behavioral dimensions. Importantly, the study showed the feasibility and scalability of participant-led biosample collection, real-time data integration, and ML-guided interpretation. These findings emphasize the value of dense, individualized data streams in capturing health trajectories invisible to conventional population-based nutritional guidelines.

POC technologies are moving beyond single-analyte readouts toward multi-analyte, networked systems coupling real-time data with ML-driven interpretation. In these envisioned architectures, the POC device becomes a bidirectional interface between the physiological state of the user and intelligent algorithms capable of contextualizing that state within an evolving body of biomedical evidence. High-frequency streams from wearables and self-reported inputs were merged with molecular snapshots obtained from the blood, saliva, or microbiome assays. These heterogeneous data are channeled into ML pipelines, often lightweight LLM backends adapted through parameter-efficient fine-tuning (e.g., LoRA and quantization). The result is a continuously updated, personalized portrait of metabolic health that can trigger immediate, tailored dietary or lifestyle recommendations.

A key element in this PN framework is retrieval-augmented generation (RAG) which can be used in conjunction with large language models (LLMs). RAG enables LLMs to query the current literature and clinical guidelines at inference time. This keeps decision support aligned with the latest evidence while enhancing transparency for clinicians, nutritionists, and users. As conversational agents, these RAG-enhanced LLM systems can translate metabolic insights into accessible coaching messages, reinforcing adherence and supporting behavior change. Continuous POC feedback loops using metrics such as glycemic variability, heart rate dynamics, or sleep quality permit LLM model refinement and finer personalization. Scalability and inclusivity are addressed through edge-optimized inference and culturally aware training corpora. The deployment of compact ML models locally reduces reliance on cloud connectivity, which is a critical consideration in low-resource settings. Meanwhile, fine-tuning ethnographically representative nutrition datasets mitigates bias and tailors guidance to regional dietary norms, genetic backgrounds, and environmental exposures.

### 5.3. Decentralized Clinical Trials (DCTs)

Traditional clinical trials often face high costs and limited patient access. DCTs address these limitations by leveraging digital technologies to boost participation rates and reduce operational expenses [139,165]. The COVID-19 pandemic further accelerated DCT adoption, making remote monitoring and data capture industry norms [166]. POC devices, such as wearables with mobile apps and LoC systems, facilitate continuous, real-world data collection: smartwatches track vital signs, mobile apps collect patient-reported outcomes, and LoC platforms deliver on-site diagnostics [33,94,135,137]. ML algorithms enhance POC datasets by predicting patient responses and supporting individualized treatment plans. DL models interpret ECG waveforms for cardiac monitoring [30,167], NLP mines electronic health records to screen trial eligibility, and predictive models optimize enrollment by forecasting treatment outcomes [168].

The integration of LLMs with RAG systems represents a promising advancement in the development of ML-augmented DCTs [169,170,171,172]. This combination enables real-time bidirectional communication between participants and research personnel, offering context-aware responses to queries about trial participation, procedures, and protocols. Consequently, reliance on in-person visits is reduced, enabling more flexible and participant-centered trial designs. Multimodal data streams, including electronic surveys, chat transcripts, voice recordings, and video assessments, can be ingested directly into the LLM-RAG pipeline [169,171]. Automated data processing minimizes transcription errors, enhances data completeness, and significantly reduces the operational costs associated with manual data entry [173]. Continuous analytics of live data feeds enables early detection of safety or efficacy signals. When data shows superior intervention performance, protocols can be modified dynamically, avoiding prolonged interim analysis. This real-time trial adaptation can accelerate timelines, reduce costs, and expedite regulatory reviews and therapeutic availability.

**Figure 6:**
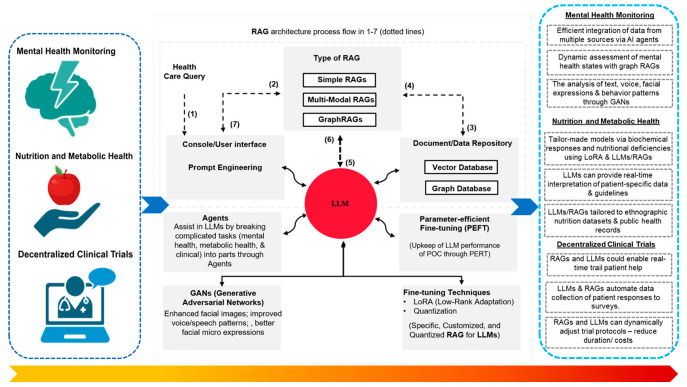
Emerging applications of machine learning in point-of-care (POC) technologies include: (1) mental health monitoring through ML analysis of speech, facial cues, and biosignals; (2) personalized nutrition guided by real-time metabolic assessments; and (3) decentralized clinical trials using mobile and wearable tools for remote monitoring. AI integration across these domains enables real-time, adaptive, and patient-centered healthcare, enhancing decision-making, engagement, and trial efficiency.

Figure 6 highlights some of the emerging frontiers in POC and AI/ML. These include AI-enhanced POC tools for mental health monitoring, PN, and DCTs, where real-time analytics, wearable integration, and LLM-powered interactivity redefine healthcare delivery. Within this emerging framework, ML-driven speech analysis offers promising avenues for remote mental health assessment, whereas wearable POC sensors provide continuous, real-time metabolic or PN monitoring. The integration of LLM-RAG conversational agents and adaptive analytics further enhances system efficiency and user engagement. Collectively, these advancements could substantially reshape clinical and healthcare delivery methodologies and expedite the move toward personalized, preventative, proactive, and precise health [168,169,171,174].

## 6. Conclusions

The integration of AI and ML into POC technologies represents a transformative shift in modern health care. These technologies now engage POC devices as sophisticated and proactive clinical decision aids, as opposed to rote and passive measurement tools. With ML technologies, real-time monitoring, predictive diagnostics, and personalized therapy become possible, thereby enhancing technology as well as improving healthcare access, efficiency, and patient-centered outcomes. DL architectures, such as CNNs and RNNs, extract patterns from biosensors, imaging, and physiological data. CNNs interpret image diagnostics and glucose measurements, whereas RNNs and LSTM networks model temporal trends in ECG and glucose readings [49,109,134]. Transformer models enhance performance by integrating multimodal data streams. Ensemble methods, such as RF and XGBoost, provide accuracy in clinical classification tasks, particularly with wearable sensors and health records. RL enables dynamic feedback for automated insulin delivery, whereas SHAP tools provide transparency in model decision-making [51].

ML-integrated POC technologies democratize healthcare by providing high-quality diagnostics in remote or underserved areas, where access to laboratory infrastructure or specialists is often limited. ML-driven glucose forecasting enables proactive patient intervention and remote decision support through interpretable hybrid learning models [49]. ML-enhanced mobile platforms, such as portable ultrasound or ECG devices, offer frontline diagnostics that are accurate, scalable, and operable by nonexpert users. Such systems reduce healthcare disparities by ensuring timely intervention and continuous care even in low-resource settings [32]. With many POC systems, ML enhances healthcare efficiency by automating time-intensive tasks, such as data interpretation, anomaly detection, and risk stratification, thereby reducing clinician workload and minimizing diagnostic errors. These efficiencies expedite treatment decisions, streamline clinical workflows, and facilitate DCTs through remote monitoring and real-time data acquisition. Simultaneously, patients benefit from adaptive alerts, behavior-aware recommendations, and greater autonomy in managing their health factors, which collectively improve adherence, engagement, and clinical outcomes.

The convergence of AI, IoMT, and cloud computing will undoubtedly enhance the capabilities and reach of POC systems. However, continued investment in algorithm robustness, data privacy, equitable model development, and clinical validation is essential. Ethical governance and user-centered design are key to ensuring that these innovations are safe, effective, and inclusive. In summary, AI and ML are not merely augmenting POC devices but are redefining them. As these technologies continue to evolve, they hold the promise of reshaping healthcare into a more accessible, predictive, and personalized system capable of meeting the needs of diverse populations around the world.

## Figures and Tables

**Table 1:** AI Algorithms used in current POC applications.

AI Algorithm	Applications in POC	Advantages	Disadvantages	References
Convolutional Neural Networks (CNNs)	Image-based sensing (hand-held ultrasound and optical glucose spectroscopy); ECG/PPG arrhythmia detection	Excellent spatial-feature extraction; well-suited to 2-D sensor outputs (images and spectrograms)	Need large labeled datasets; GPU/TPU requirements can be prohibitive on edge devices	[9,134]
Recurrent Neural Networks/LSTM	Continuous-glucose monitoring (CGM) forecasting; sequential ECG analysis	Capture long-range temporal patterns in physiological streams	Susceptible to vanishing gradients; output quality degrades with noisy or missing data	[45,49,100,125]
Transformer and Hybrid Attention Models	Multimodal signal analysis (CGM + activity + sleep); real-time ultrasound interpretation	Attention lets model focus on salient; irregular segments; handles asynchronous inputs	Large memory/computation footprint; data-hungry	[32,66]
Random Forest (RF)	Tabular risk-stratification dashboards; blood-glucose level prediction	Robust to outliers and missing values; fast inference on CPUs	May overfit noisy signals; interpretability moderate	[46,65,72]
XGBoost/Gradient-boosted trees	CGM trend prediction; portable vital-sign monitors	High accuracy/speed; native handling of missing data	Hyper-parameter tuning is complex; sensitive to signal noise	[49,59]
Reinforcement Learning (RL)	Closed-loop insulin delivery (‘artificial pancreas’); adaptive wearable dosing assistants	Learns optimal actions from feedback; personalizes over time	Safety-critical training; needs extensive simulation/data	[24,66,106]
Explainable AI (e.g., SHAP)	Post-hoc interpretation of CGM/ECG models; clinician-facing dashboards	Quantifies feature impact → boosts trust and regulatory acceptance	Adds compute overhead; explanations are approximations	[51,52,53]
Unsupervised/Clustering methods	Patient stratification in mobile diagnostics; anomaly detection in wearable streams	No labels required; uncovers hidden phenotypes	Validation and clinical mapping can be difficult	[43]

**Table 2:** Overview of commercially available AI/ML-powered POC devices with their application areas, algorithms, validation status, and key features.

Device (Maker)	Application Area	Algorithm Type (Indicative)	Clinical Validation and Regulatory Status	Notable ML/POC Features	References
FreeStyle Libre 3 (Abbott)	Diabetes: continuous glucose monitoring (CGM)	Time-series forecasting and predictive analytics; CGM forecasting commonly uses LSTM/RNN, transformers, and gradient-boosted trees in POC settings.	Interventional study (2024–25): MARD vs. venous YSI ≈ 11.6%; studies show device-dependent differences in TIR/GMI across CGMs (Libre 3 vs. G7 vs. Simplera).	Real-time Bluetooth low energy (BLE) streaming to phone; ML-enabled trend prediction and alerts via paired apps; supports proactive insulin decisions.	[110,111]
Dexcom G7 (Dexcom)	Diabetes: CGM	Time-series forecasting (as above)	Interventional study: MARD ≈ 12.0%; FDA iCGM clearance Dec 2022; expanded 15-day wear cleared Apr 2025.	Integrated decision support via mobile; predictive alerts.	[24,110,111,112]
Simplera/Simplera Sync (Medtronic)	Diabetes: CGM (incl. AID use)	Time-series forecasting (as above)	Interventional study: MARD 11.6%; performance gains in hypoglycemia vs. comparators; CE-mark 2023; FDA Aug 2024; Simplera Sync approved Apr 2025 for 780G AID.	Parallel-wear studies show clinically meaningful therapy metric differences vs. peers (TIR/GMI).	[110,111,113]
Butterfly iQ/iQ+/iQ3 (Butterfly Network)	Portable ultrasound (POCUS)	Deep learning (CNNs) for image quality guidance, classification, and feature extraction	RCT in ED (n = 110): sensitivity 92.9%, specificity 92.3%, accuracy 92.5% vs. cart systems; ophthalmic triage: 90% sens/95% spec (κ = 0.85); prehospital lung US improved AHF diagnosis and treatment time; FDA 510(k) for iQ3 (4 January 2024); Auto B-Line Counter AI cleared.	Smartphone-tethered probe; real-time AI guidance; expanding SaMD-aligned toolset for non-expert use.	[118,119,120,121,122,123]
Apple Watch (Series 4+) (Apple)	Wearable cardiac and sleep monitoring	On-device arrhythmia classification (single-lead ECG), PPG analytics; CNN/ML classification are widely used for ECG/PPG in POC	Post-op cohort: AFib detection 73.9% sens/95.7% spec vs. telemetry; ECG app 95% sens/spec for AFib; AFib History: FDA-cleared (2022), qualified 2024, weekly AF burden within ±5% of reference; Sleep Apnea (2024) 510(k): ~66% sens, >95% spec vs. PSG.	Consumer-grade wearable with medical features; passive monitoring + clinician-shareable outputs.	[98,127,128,129]
Swift Skin (Swift Medical)	Wound assessment and documentation	ML-assisted computer vision for wound sizing/composition; standardized color/size via fiducial marker	Time-motion study: ~79% faster assessments; higher first-attempt image quality (92.2% vs. 75.7%); saves 1–2 min/assessment; uses FDA-registered HealX marker; improves documentation accuracy and early complication detection.	Mobile imaging with calibration; workflow and quality gains in chronic wound care.	[132,133]

**Table 3:** Gaps and challenges in current POC and AI integration.

Category	Challenges	Description	Impacts	Example Applications Affected	Suggested Solutions	References
Technical and Performance Limitations	-Degraded accuracy in real-world settings	-AI systems perform poorly outside controlled environments due to noise, sensor drift, and physiological variability.	-Reduces reliability in sensitive diagnostics such as metabolic, cardiac, or mental health monitoring.	-Wearables, portable ECGs and glucose monitors	-Adaptive algorithms, robust sensor calibration, and noise filtering	[45,46,48,101,103,110,134]
Data and Model Limitations	-Limited generalizability -Poor data quality	-ML models trained on narrow datasets perform poorly on diverse populations. -Missing values, irregular sampling, and noisy input degrade AI reliability.	-Biased predictions; poor performance in global/underserved settings. -Unstable outputs; reduced model trustworthiness.	-Global health tools and mobile diagnostics -Continuous glucose monitors, wearable biosensors	-Diverse training datasets, data augmentation, and fairness audits -Signal correction, imputation methods, and data preprocessing pipelines	[32,69,134]
Ethical, Legal and Regulatory Issues	-Privacy and bias concerns -Legal ambiguity	-Sensitive data handling and biased training data raise ethical risks. -Unclear responsibility for AI-assisted decisions in clinical care.	-Disparities in outcomes; reduced user trust and legal complications. -Deployment hesitancy in regulated environments.	-Mental health apps and biometric wearables -AI diagnostic assistants, automated triage systems	-Differential privacy, bias audits, and transparent model reporting -Legal frameworks, shared accountability models	[117,138]
Usability and Clinical Integration	-Low clinician trust -Workflow disruption	-Opaque AI models (black-box) hinder clinical acceptance. -High cognitive load, poor UI/UX, and lack of training limit clinical integration.	-Slow adoption despite accuracy benefits. -Inconsistent use; potential errors or delays.	-Medical imaging AI and predictive monitoring -Hospital-based POC systems and remote monitoring dashboards	-Explainable AI (e.g., SHAP and LIME), clinician co-design -Training programs, simplified interfaces, human factors design	[51,52,53,135,136]
Scalability and Access	-Infrastructure dependency -High cost	-AI-enhanced POC tools rely on stable power, internet, and specialized hardware. -Advanced POC AI systems are expensive to deploy and maintain.	-Limited usability in low-resource settings. -Hinders mass adoption and democratization.	-Smart wound sensors and edge AI diagnostics -Smart diagnostics in rural clinics, mobile labs	-Offline/edge AI models, solar-powered devices -Model compression, open-source platforms, and government subsidies	[32,59,64,131,139]
Sensor and Device-Specific Issues	-Signal interference and inconsistency -Wearable sensor limitations	-Sensors suffer from contamination, fouling, lighting variation, or user handling errors. -Battery life and algorithm inefficiency limit continuous use.	-Inaccurate AI predictions; poor reproducibility. -Interrupted monitoring; inconsistent results.	-Electrochemical biosensors, optical imaging, microfluidics -Wearables for heart rate, glucose, and motion	-Sensor design improvements, robust preprocessing, and user guidance -Energy-efficient AI, hardware optimization, and lightweight models	[17,74,81,97,137,140]

## Data Availability

No new data were created or analyzed in this study.

## Abbreviations

The following abbreviations are used in this manuscript:

AI	Artificial Intelligence
CDSS	Clinical Decision Support System
CGM	Continuous Glucose Monitoring
CGMs	Continuous Glucose Monitors
CNN	Convolutional Neural Network
DCTs	Decentralized Clinical Trials
DL	Deep Learning
ECG	Electrocardiogram
GRUs	Gated Recurrent Units
IoMT	Internet of Medical Things
LLM	Large Language Model
LSTM	Long Short-Term Memory Networks
ML	Machine Learning
NLP	Natural language processing
PCA	Principal Component Analysis
PN	Precision Nutrition
POC	Point-of-Care
POCUS	Point-of-Care Ultrasound
PPG	Photoplethysmography
RAG	Retrieval-Augmented Generation
RF	Random Forests
RL	Reinforcement Learning
RNN	Recurrent Neural Network
SVM	Support Vector Machine
STFT	Short-Time Fourier Transform
t-SNE	t-distributed Stochastic Neighbor Embedding
